# Process evaluation of the 90-90-90 targets of surge project in Addis Ababa, Ethiopia: a case study evaluation

**DOI:** 10.1186/s12913-023-10415-9

**Published:** 2024-01-02

**Authors:** Belete Kefyalew Eshetu, Tesfahun Zemene Tafere, Geta Asrade, Tsegaye Gebremedhin Haile

**Affiliations:** 1 Communicable Disease Control Project, Addis Ababa Health Bureau, Addis Ababa, Ethiopia; 2 Department of Health Systems and Policy, Institute of Public Health, College of Medicine and Health Sciences, University of Gondar, P.O. Box: 196, Gondar, Ethiopia

**Keywords:** Evaluation, Availability, Compliance, Accommodation, Fidelity, HIV, Surge project, Ethiopia

## Abstract

**Background:**

Human Immunodeficiency Virus (HIV) is a major public health problem that continues to pose an enormous challenge to mankind’s survival worldwide. In urban Ethiopia, the HIV prevalence among adults aged 15–49 years is 2.9%, while in Addis Ababa, it is 3.4%. To take the edge off, the Ethiopian government has been implementing the 90-90-90 strategy also known as the surge project, in urban cities. However, the implementation of the program has not been evaluated. Thus, we evaluated the process of the 90-90-90 targets of the surge project in Addis Ababa, Ethiopia.

**Methods:**

We conducted a case study with concurrent mixed-methods evaluation. We used indicator-driven evaluation dimensions –availability and accommodation dimensions from the health services access and compliance and fidelity from implementation fidelity frameworks to test the program process theory with a total of 52 indicators. We interviewed a total of 419 clients and 210 healthcare providers and reviewed 417 clients’ cards and 17 registries. We also conducted 30 key informant interviews and resource inventory. A binary logistic regression analysis was done to identify factors associated with clients’ satisfaction. We transcribed and translated the qualitative data and analysed thematically. Finally, we judged the overall process of the surge project based on the pre-seated judgmental criteria as; needs urgent improvement, needs improvement and well implemented.

**Results:**

We found that 90% of the project process was as per the program process theory measured by the availability of resources (95.8%), compliance (88.0%), fidelity (84.7%), and accommodation of services (89.3%). We found a shortage of human power, test kits, and viral load testing machines. The commitment of health care providers, provider-client interaction, and clients’ satisfaction with the service at card rooms were found to be poor. Moreover, being aged 15–24, being married and government government-employed were negatively associated with clients’ satisfaction with antiretroviral therapy services.

**Conclusion:**

The process of the surge project needs improvement. Moreover, the achievements of the first two 90–90 targets were poor. Therefore, implementers need to take intensified action for the availability of resources and to improve the commitment of healthcare providers through refreshment training.

## Background

Human Immunodeficiency Virus (HIV) continues to be a major public health concern worldwide [1]. In East Africa, 19 million people are living with HIV. Among them, 960,000 are newly infected per year. Approximately 54% of those infected have access to antiretroviral therapy, and 59% of children are on antiretroviral treatment [2]. HIV continues to be a serious international health concern, and sub-Saharan Africa remains the most affected region, accounting for over 25.6 million people currently living with HIV [3, 4]. As a result, HIV remains a challenge to achieve the universal access target and ensure the quality of HIV/AIDS care and treatment services in many low-income countries hardest hit by HIV epidemics [5].

The burden of HIV prevalence among women and men aged 15–49 years in urban Ethiopia is 2.9%, which ranges from less than 0.1% in Somalia to 4.8% in Gambela and 3.4% in Addis Ababa [6]. Only 65.2% of people living with HIV knew their status, while 63.3% were on Antiretroviral Therapy (ART) and 58% were viral load suppression in Addis Ababa in 2018 [7].

In 2014, the Joint United Nations Programme on HIV/AIDS (UNAIDS) and partners launched an ambitious but achievable treatment target [8]. These targets called for 90% of all people living with HIV to know their HIV status, 90% of all people with diagnosed HIV infection to receive sustained antiretroviral therapy (ART), and 90% of all people receiving antiretroviral therapy, irrespective of immune status, to achieve viral suppression by 2020. These goals are crucial steps in ending the AIDS epidemic by 2030 [9].

The UNAIDS recommends that the share of HIV-related clinical services provided in community settings must rise from 5% in 2016 to 30% to achieve the 90-90-90 targets. This can be accomplished by improving the number of people receiving ART, strengthening the health system approach, providing training, empowering community health workers to perform ART-related tasks, and bringing services closer to the people who need them over the next five years [10].

Ethiopia has developed a nation-wide five-year HIV/AIDS strategic plan (2015–2020) based on the UNAIDS 90-90-90 target, to end the AIDS epidemic by 2030 [11]. To achieve this ambitious target, the country initiated a surge project in 2017, which is currently being implemented in 20 priority towns across the country [11]. This project serves as a robust movement towards achieving the 90-90-90 national target, focusing on the identification of new HIV-positive individuals, ensuring their access to care and treatment, and facilitating viral load suppression for those living with HIV. While prior research in Ethiopia has mainly concentrated on HIV/AIDs prevalence [12, 13], there has been limited attention given to understanding the implementation of the surge project, including its inputs, activities, and outputs, as well as the facilitators that may facilitate or hinder its process. As a result, we evaluated the process of the surge project to produce comprehensive evidence about its implementation as well as identify the facilitators and barriers to inform decision-making. Our findings will provide valuable insights to policymakers and program managers, enabling them to enhance the implementation of this crucial aspect of healthcare.

## Methods

### Evaluation design and settings

We conducted a case study evaluation with mixed-methods in Addis Ababa, the capital city of Ethiopia, in the case of Lideta sub-city. The sub-city has two governmental health institutions, 28 private health institutions, two supporting local non-governmental organisations (NGOs), and a total population of 214,796. The surge project for the HIV response, aiming to achieve the 90-90-90 targets, is being implemented in this sub-city. It involves one governmental hospital and three governmental health centres, collectively serving more than 157,000 clients per year.

The study participants were (a) ART clients aged 15 years and above who were scheduled in the ART clinic, (b) health care providers working in the implementing health centers for more than two years, (c) health facility managers, (d) surge project supportive staffs, and (e) ART documents.

### Evaluation approach and dimensions

**Fig. 1:**
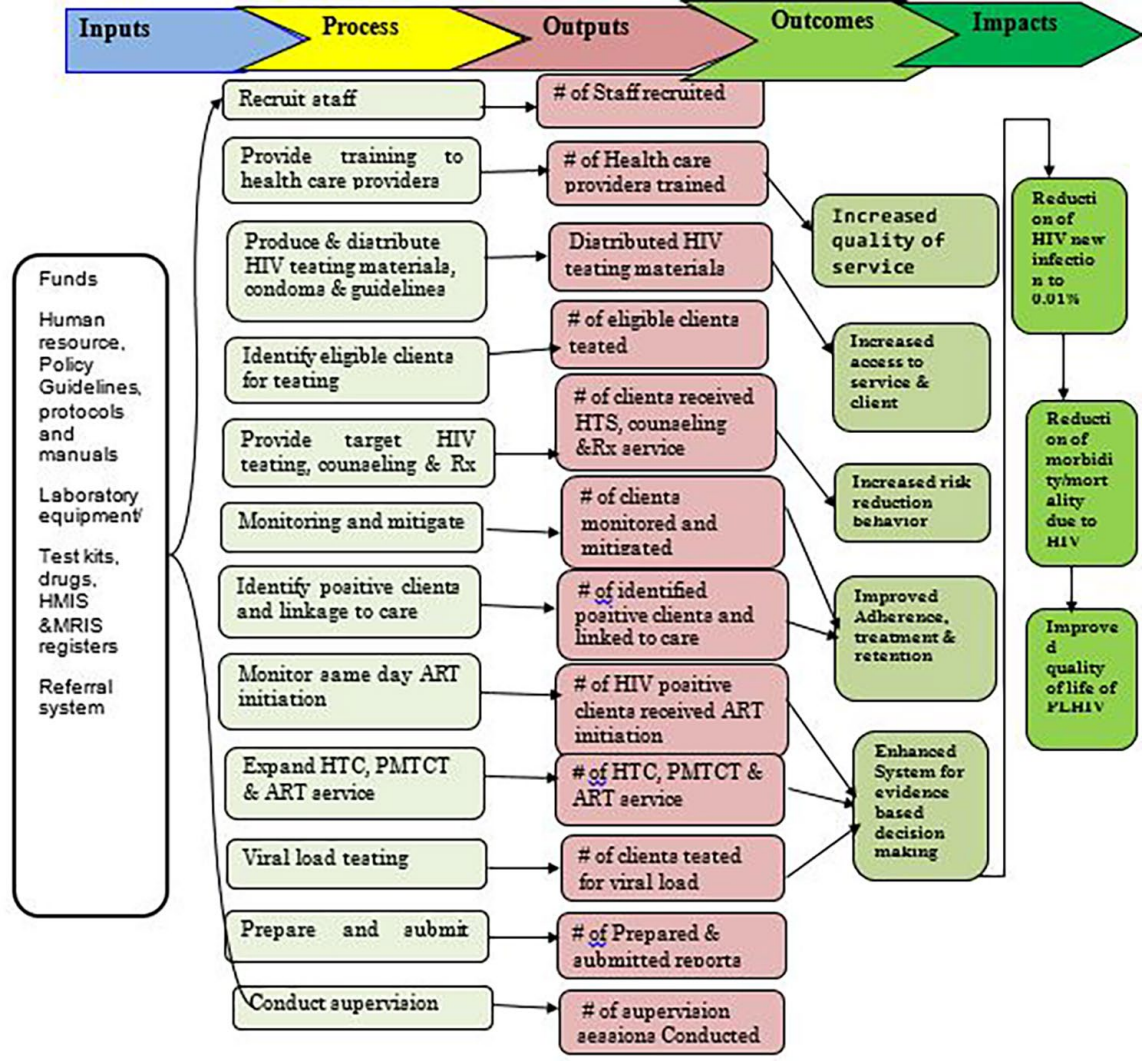
A logic model of the 90-90-90 targets of surge project in health centers of Lideta sub-city, Addis Ababa, Ethiopia, 2020

We used a formative approach to evaluate the process theory of the surge project implementation. The program’s process theory comprises the program’s organisational and services utilisation plan [14]. As a result, the organizational structure of the program was developed from the perspective of program management, considering both the tasks and activities that the program is anticipated to undertake, as well as the required resources. The service utilisation plan, on the other hand, concentrated on the crucial assumptions regarding how and why the intended recipients of the project services engaged with the program and continued until they received sufficient services to initiate the change process described in the program impact theory. The overall project description is presented in Fig. 1. In our process evaluation, we focused on the process and we used the availability and accommodation dimensions from the access framework [15] and, compliance and fidelity dimensions from the implementation fidelity framework [16].

We collected the quantitative and qualitative data simultaneously, analysed them separately, and integrated during the interpretations of findings phase.

### Sample size and sampling procedures

The sample size for the exit interview to measure the accommodation of the surge project was calculated using a single population proportion formula: ${\rm{n}}\,{\rm{ = }}\,\frac{{{\rm{z}}{{\left( {\frac{\alpha }{2}} \right)}^2}\,{\rm{x}}\,{\rm{p}}\,(1 - {\rm{p}})}}{{{{\rm{d}}^2}}}$ with the assumption of a 5% margin of error (d), 95% confidence level, *p* = 50%, and 10% non-response. The final calculated sample size was 422. Then, we did a proportional allocation to health centers (HC) based on the total number of ART users in each health center.

We did a total of 48 and 42 direct non-participatory observations for Standard Operating Procedures (SOP) and provider-client interaction, respectively, and 2 years of DHIS reports, and 417 ART clients’ cards were reviewed for measuring the compliance and fidelity dimensions, retrospectively. We inventoried three governmental health centres. Moreover, thirty key informants were purposively selected and interviewed.

We used a systematic sampling technique to select the study participants at the ART clinic. Those key informants and ART clients who were severely ill and unable to have an interview were excluded from the interviews.

### Variables and measurements

Evaluation dimensions are measurable aspects of program performance that an evaluation intends to examine [17]. Availability refers to the physical presence of services, drugs, equipment, qualified medical personnel, guidelines, registers, and infrastructures [15]. We assessed the availability of resources using 19 indicators to determine whether essential drugs, medical equipment, and infrastructures required for the project were available or not. Compliance is the practice of HIV testing, care, and treatment services based on the national consolidated guidelines for comprehensive HIV prevention, care, and treatment [18, 19]. We measured the compliance dimension using 10 indicators, which included assessing the adherence of health facilities and healthcare providers to the guidelines in delivering HIV testing services (HTS) and ART services. Fidelity refers to the degree to which programs are implemented as intended by the program developers [19]. We measured the fidelity of the surge project using 12 indicators which included assessing the adherence, coverage, and quality of HTS and ART services delivery.

Moreover, we used 11 indicators to measure the accommodation dimension. Accommodation refers to the setup of the organization to accept clients, and the clients’ convenience of its appropriateness, as well as the ability to accommodate organizational factors [15]. Similarly, we measured the satisfaction of clients with the accommodation of ART services using 11 indicators, assessed through a five-point Likert scale ranging from (1 = very dissatisfied to 5 = very satisfied). Accordingly, the minimum score for the 11 indicators was 11 and the maximum was 55, the mean score was calculated and found to be 47. Thus, clients who scored above the mean were considered satisfied, while those who scored at or below the mean were considered dissatisfied. These indicators were developed and adapted from the WHO consolidated guidelines on the use of antiretroviral drugs for treating and preventing HIV infection and other related literature [20, 21]. The indicators’ weight was determined by stake-holders during the evaluability assessment. The weight was calculated using the formula $\left( {\frac{{{\rm{Observed}}\,{\rm{number}}\,{\rm{x}}\,{\rm{Indicator}}\,{\rm{weight}}}}{{{\rm{Expected}}\,{\rm{number}}}}} \right)$. The achievement was calculated by $(\frac{\text{S}\text{c}\text{o}\text{r}\text{e}}{\text{W}\text{e}\text{i}\text{g}\text{h}\text{t}}\text{x}100$). Then, the judgment parameter for the indicators was rated as < 75, 75–90, and > 90 as poor, good, and very good, respectively.

### Data collection tools and procedures

We used a structured questionnaire for the exit interviews, an observation and resource inventory checklist, and guiding questions for key informant interviews. The questionnaires were adapted from the surge project monitoring and evaluation documents and other related literatures [22, 23]. These tools were initially developed in English and then translated to the local language (Amharic) and back to English to ensure clarity and consistency. For data collection, we recruited two BSC health officers and one BSC nurse as data collectors, and one BSC nurse a supervisor from nearby health centers. Before commencing the data collection process, the data collectors and supervisors underwent a one-day training on data collection methods and tools. The entire data collection process was closely supervised daily. In cases where the collected data were incomplete or invalid, it were rejected to maintain the quality of the evaluation findings.

We conducted a pre-test with 21 participants, which constituted 5% of the total sample, and necessary modifications and amendments were made. Moreover, the Cronbach alpha value of 0.81 demonstrated the reliability of the tools used for collecting quantitative data. For qualitative data, we conducted the interview after debriefing key informants and arranging suitable times and locations for the interviews. The quality of qualitative data was ensured by taking daily notes during key informant interviews. Exit interviews were conducted in the waiting room to ensure both visual and auditory privacy for the clients. Likewise, non-participatory direct observation was conducted while healthcare providers were delivering services to the clients.

Each observation and key informant interview were carefully recorded on the field notes. Both the supervisor and the principal evaluator checked the accuracy of the data collected by the data collectors through randomly selected indicators. Additionally, reports, health management information system (HMIS) registers, feedback, and performance meeting minutes from the past two years were reviewed retrospectively.

### Data management and analysis

The quantitative data were cleaned and checked for completeness and consistency, coded and entered into Epi-data version 4.4.1 and exported to SPSS version 20 for analysis. Univariate descriptive analysis was computed, and results were presented using tables and narrations. A binary logistic regression analysis model was done to identify factors associated with clients’ satisfaction and variables with a *p*-value of less than 0.25 during bi-variable analysis were the candidates for multivariable logistic regression. In multivariable logistic regression, those variables having a *p*-value of less than 0.05 and adjusted odds ratio (AOR) with 95% confidence intervals (CI) were considered statistically significant factors of satisfaction. Model goodness-of-fit was checked using the Hosmer Lemeshow test, which resulted in 88.2%, indicating that the model fitness was good.

The audio-recorded qualitative data were transcribed and translated into text format, then coded and categorized through a thematic analysis approach. Both qualitative and quantitative data were mixed and integrated during the interpretation phase, supplementing the quantitative findings.

The weight of each evaluation dimension and the respective indicators used to assess the performance of the surge project for the 90-90-90 targets of HIV response was determined through stakeholder agreement, based on the degree of relevance. Accordingly, each dimension was judged as poor, good, and very good if the score was < 75, 75–90 and > 90, respectively.

The scores of each evaluation dimension were aggregated to determine the overall level of performance of the surge project, based on the predetermined judgment criteria. The final assessment of the surge project’s process was categorised as needs urgent improvement, needs improvement, or well-implemented if the score was < 75, 75–90, and > 90, respectively.

## Results

In our evaluation, we conducted a survey among a total of 419 ART clients (with a response of 99%). We involved 210 healthcare providers, conducted resource inventory in 1 hospital and 3 health centers, observed 42 provider-client interaction sessions, reviewed 417 client’s cards and 17 registers for document review. Additionally, we conducted 30 key informants’ interviews.

### Availability of resources

We found that a total of 220 healthcare workers were available in the health centers, of whom 48% were nurses and 1.4% were medical doctors. Nearly 92% of the healthcare providers are trained on the 90-90-90 surge project as per the recommended protocol (Table 1). From the qualitive analysis, we also found that the majority of the healthcare providers are trained on the surge project. This is supported by a 35 years old disease prevention team leader key informant’s response as.

“*Even though only 34 health care providers received comprehensive HIV counseling and testing, we gave on-the-job training for all health care providers to facilitate the surge project activities in each SDP. Training is not our challenge rather the shortage of manpower*“ [a 35 years old female disease prevention team leader].

**Table 1:** Availability of trained health care providers for the 90-90-90 targets of the surge project in Addis Ababa, Ethiopia

Services	Profession category	Minimum expected number	Trained n (%)	Duration of training in days
Comprehensive ART	Health officers	6	6 (100.0)	12
	Nurse	18	18 (100.0)	
Comprehensive PMTCT	Midwives	29	22 (75.9)	15
Comprehensive HIV counselling and testing	Health officers	8	8 (100.0)	21
	Nurse	12	12 (100.0)	
Risk Assessment utilization, PITC, HTS, and 90-90-90 related activities	Health officers	29	29 (100.0)	1
	Nurse	106	95 (89.6)	
	Midwives	29	26 (89.7)	
Viral load sample collection and testing	Lab. Technologist	13	13 (100.0)	5
	Lab. Technician	6	6 (100.0)	

We found that all essential drugs and medical supplies were in stock whereas the dried blood spot (DBS) kit and Cotrimoxazole suspension were stocked out in the last six months in all health centers. This is also supported by our qualitative finding in which there is a shortage of some test kits and testing reagents; as supported by one of the key informant’s responses.

“*There was a shortage of test kits for two weeks in June, one week in July, and three weeks in September 2019. Due to this issue, we were unable to achieve our testing targets. Additionally, there was a shortage of INH from July to October 2019. Despite reporting a stockout of DBS kits since August 2019, the problem remains unresolved. There have been no shortages of laboratory equipment, materials, and chemical reagents in the last six months. However, viral load testing reagents and machines were not made available in the health centres* ” [A 32 years old male BSC nurse].

We found that guidelines were available in ART, PMTCT, and VCT clinics, but other PIHTC-delivered SDPs had no guidelines; instead, they relied on testing algorithms and standard operating procedures (SOPs) or protocols based on the national guidelines. About 82.4% of healthcare providers received specific guidance about the importance of confidentiality and adhering to the national guidelines for ART and related activities. This is supported by our qualitative findings; the majority of the healthcare providers received training on adhering to guidelines and keeping confidentiality for clients. A key informant’s response supported this;

“*We received orientation and on-the-job training performing HIV testing services and maintaining confidentiality. While guidelines did not exist in OPDs, manuals, standard operating procedures, algorithms, and risk assessment tools were available. Consequently, we used the risk assessment tool to identify eligible clients for HIV testing and followed the manuals and algorithms for testing procedures. However, some providers faced challenges in using the risk assessment tools properly*” [A 30 years old male health officer].

We found that the majority of job aids and infrastructures were available (Table 2). Moreover, we found from the qualitative analysis that some of the job aids are available, but there is a shortage of registries and data storage hardware. A key informant replied as;

“*When the project began, the Regional Health Bureau promised to deliver a laptop for monitoring and evaluation officer, one printer, one shelf, three chairs, three tables, and one desktop for each service unit of the health centres. However, the bureau only fulfilled the shelves, chairs, and tables. To avoid interruptions in reporting and other computer-related activities, we resorted to using the health facilities’ old computers and printers”* [A 26 years old female key informant from the health centre].

**Table 2:** Availability of job aids and infrastructures of the 90-90-90 targets of the surge project in Addis Ababa, Ethiopia

Job aids and infrastructures	Available at the time of observation	Available in the last six months
	n (%)	n (%)
Testing SDPs	48(100)	48(100)
Adherence counseling rooms with auditory and visual privacy	3(100)	3(100)
Service delivery points available for HIV testing	48(100)	48(100)
Tables	12(100)	12(100)
Chairs	12(100)	12(100)
Printers	0	0
Photocopy machine	0	0
Computers	2(67)	2(67)
Laptops	0	0
ART Smartcard database	3(100)	3(100)
DHIS2 database	3(100)	3(100)
PTQIT database	3(100)	3(100)
Shelf	3(100)	3(100)
Telephone phones	3(100)	3(100)
Weight scale	6(100)	6(100)

During our observation, the risk assessment tool was available in each adult and paediatric OPDs, although not sufficiently printed. To solve this issue, HIV testing eligibility criteria were displayed on the wall of each SDPs in one health centre but not others. Furthermore, there were safe rooms for HIV testing and counseling in ART and VCT, each equipped with a window and door. Healthcare providers also ensured auditory and visual privacy while providing services to clients. However, in other service delivery points, there were no separate counseling and testing rooms. Hence when one provider counselled and tested the client, other clients within the same room lacked privacy, their consultations were visible and audible to others.

We found that the overall availability score of the 90-90-90- targets of the surge project was 95.8% (Table 3).

**Table 3:** Resources availability judgment for the 90-90-90 targets of the surge project in Addis Ababa, Ethiopia

Indicators	E	O	W	S	A (%)	JP
The proportion of health care providers trained on the surge project services	164	150	6.8	6.2	91.5	V.good
The proportion of health care providers trained on comprehensive ART	24	24	6.6	6.6	100.0	V.good
Percentage of health care providers trained in compressive PMTCT	29	22	4.9	3.7	75.9	Good
The proportion of health care providers trained in compressive HIV testing and counseling (VCT)	20	20	6.2	6.2	100.0	V.good
The proportion of laboratory professionals trained in viral load sample collection and testing	19	19	5.9	5.9	100.0	V.good
The proportion of PITC-provided SDPs in the health centers	48	48	5.6	5.6	100.0	V.good
The proportion of adherence counseling room with visual and audio privacy	48	42	6.2	5.4	87.5	Good
The proportion of relevant national guidelines, manuals, and treatment charts in ART and PMTCT clinics	12	12	5.6	5.6	100.0	V.good
The proportion of relevant PITC protocol and algorithms in each SDP	48	40	3.6	3	83.3	Good
The proportion of relevant risk assessment tools in each SDP	21	21	4.9	4.9	100.0	V.good
The proportion of test kits pcs in stock for the last six months	9165	8988	4.2	4.1	98.0	V.good
Percentage of EDTA tubes in stock for the last six months	625	625	4.9	4.9	100.0	V.good
Percentage of Nuc- tubes in stock for the last six months	632	632	4.8	4.8	100.0	V.good
The proportion of OI drugs in stock for the last six months	100	100	4.8	4.8	100.0	V.good
The proportion of ARV drugs in stock for the last six months	18,440	18,440	5.7	5.7	100.0	V.good
The proportion of cotrimoxazole (CT) 960 mg and 240 mg per 5ml in stock for the last six months	7020	6300	4.7	4.2	89.7	Good
The proportion of isoniazid preventive therapy (IPT) 300 mg and 100 mg drugs for the last six months	4360	3750	5.8	4.9	86.0	Good
The proportion of job aids in the last six months	51	41	4.6	3.7	80.4	Good
The proportion of database (ART Smart Care, PTQIT, and DHIS) in the last six months	9	9	4.2	4.2	100.0	V.good
Overall availability score			100	95.8	95.8	V. good

E: Expected, O: Observed, W: weight, S: Score = ((observed X weight)/Expected), A (%): Achievement in percentage = ((S/W) * 100), JP: Judgement Parameter

### Compliance and fidelity to the guideline

During the direct observation, all of the ART clients were screened and assessed based on the follow-up form. Moreover, measuring clients’ weight, assessing WHO staging and assessing opportunistic infections were observed to be practised as per the recommended protocol.

Likewise, healthcare providers adhered fully to screening to co-trimoxazole preventive therapy, assessing patients’ ARV adherence and setting HIV prevention plans.

On the contrary, only 85.7%, and 19.0% of ART clients were assessed for pregnancy status and adverse drug effects (ADEs).

In the direct observation of risk assessment utilization, healthcare providers have assessed two-thirds of clients whether they have ever been tested for HIV, occupational risks and clinical signs and symptoms of HIV/AIDS. Furthermore, assessing TB and sexual risk factors of clients were the list practised which was 38.0% (Table 4).

**Table 4:** Direct observation results of the 90-90-90 targets of the surge project in health centers of Lideta sub-city, Addis Ababa, Ethiopia, 2020(n = 21)

Tasks for ART clients	Performance n (%)
**Observation of ART provider-client interaction at ART clinics**	
Measured client’s weight	21 (100.0)
Assessed the client’s pregnancy/family planning method	18 (85.7)
Assessed ART client’s functional status	19 (90.5)
Assessed WHO staging	21(100.0)
Screened for TB	20 (95.2)
Assessed opportunistic infections	21(100.0)
Assessed pain	18 (85.7)
Screened for co-trimoxazole preventive therapy	21(100.0)
Assessed their ARV adherence	21(100.0)
Set HIV prevention plan	21(100.0)
Assessed adverse drug effects (ADEs).	4 (19.0)
**Direct observation of risk assessment utilization at OPDs**	
Ask the patient ever been tested for HIV	14(66.7)
Assessed occupational risks	14(66.7)
Ask about their marital status and risk for HIV	10(47.6)
Assessed clinical signs and symptoms of HIV/AIDS	14(66.7)
Assessed STI, TB or sexual risk factors	8(38.0)

Healthcare providers were interviewed on their general attitude about the provision of the 90-90-90 targets of surge project implementation. A total of 210 healthcare providers were interviewed. All of them agreed that the surge project was a very important project to achieve the three 90 targets at the facility and regional levels. On the contrary, about one-fourth (26.0%) of healthcare providers responded that the provision of the surge project had influenced them to limit providing other routine services.

The majority of key informant interviewers argued that they did not use HIV risk assessment tools as adequately as possible.

*“Some health care providers could not use the risk assessment tool properly which means they filled the tools after the clients had left only to answer their accountability. Because of this, even if we could test a large number of clients, new HIV-positive identification was under our plan”* [A 35 years old male partner notification service officer].

Regarding the performance of positive identification (first 90), the evaluation revealed that the average HIV-positive identification performance of the health centers against their target was only 48.0%. Moreover, forty-eight service delivery points have provided HTS based on the national guidelines. HMIS, three ART and pre-ART, three positive tracking registers, and two years of monthly and weekly reports were also reviewed retrospectively. The retrospective reviews revealed that the achievement of HIV testing service was 100.0% against their target of which 1.5% were newly HIV positive.

The findings of the key informants’ interviews indicated that to achieve the first 90 targets, they developed a micro plan, strengthened and expanded HTS activities in each SDP, and monitored and mitigated missed opportunities at high-yield entry points.

Similarly, providing timely feedback to the health care providers, identifying and monitoring family members’ HIV testing status for index cases, strengthening post-test counseling, and supporting sites to track referrals were found to be the main activities justified by the key informants. They also monitored risk assessment tools and rapid test kit utilization at sites and reported weekly and monthly to facility management and the sub-city health office. But, because of the exaggerated plan, the performance of the first 90 was low.

*“Since professional staff is scarce in the outpatient department (OPD), HIV risk assessment tools were not used well and individuals who were supposed to undergo the test could never have it. Therefore, in order not to let this happen, Case Detection Officer was assigned to take care of the issue for the last six months. In paediatrics, the number of under five children seeking treatment and the professionals needed are not proportional and this in turn gave rise to less HIV risk assessment tools utilization, testing, and positive case identification. The aforementioned ones and the exaggerated plan were contributing factors to our low-performance score. Whatever the case may be, the project ignites us to strive more and I believe the project is better to be sustained”* [A 35 years old female disease prevention team leader].

*“We were given training on HIV risk assessment tools for HIV-positive identification and the rest of the second and third 90s. As a result, we have been doing more than in the past. We had also been trying hard to convince clients to know their HIV status. Even though there are additional burdens, we will use our potential and do more to achieve its objective”* [A 31 years female health officer].

Healthcare providers had a low fidelity rate of HIV risk assessment utilization and service provision as shown by the reviewed risk assessment tools utilization. This result was supported by a KII participant.

*“Even though there was the availability of HIV risk assessment tools in an emergency, it was difficult to give HIV test counseling using the tools as high trauma cases of unstable patients due to alcohol drunk come to the facilities. In addition, when health providers from different SDPs were provided with the service in an emergency on the weekend, night, and holiday, those providers would be new to the working setup, hence they do not use the HIV risk assessment tools and would not deliver testing services even though the client was eligible for HIV testing. Sometimes, health care providers also missed eligible clients by their negligence or low commitment”* [A 33 years old male emergency coordinator].

*Another KII participant also said “We oriented the health care providers if clients presenting with symptoms or signs of illness possibly attributable to HIV based on the risk assessment tools, it is a basic responsibility of health care providers to recommend HIV testing and counseling as part of routine clinical management. But they could not use the risk assessment tools properly and even they missed while clients were eligible for HIV testing. Therefore, our positive identification achievement was under the plan for the last two years”* [A 30 years old female KP/PS].

*“Despite our better HIV positive identification, the target plan was exaggerated that it was difficult to achieve this target plan as expected and clients were refused for HIV testing even they were eligible”* [A 40 years old female VCT focal].

In this evaluation, it was seen that TB clinics contributed a high positivity yield (8.3%) among all health centers whereas paediatrics OPD had no positivity yield of contributions. Furthermore, ICT (Index Case Testing), cough OPD and VCT had a positive yield contribution of 4.9%, 2.6%, and 2.5% respectively in the Lideta sub-city. Regarding population category, positivity yield was also high among partners of people living with HIV (7.7%), prisoners (6.7%), and orphans and vulnerable children (OVC) (6.7%) and the lowest in the general population (1.2%) of yield (Table 5). Overall, 406 newly positive people were identified from October 2017 to September 2019 in all health centers implementing the surge project in the Lideta sub-city. Nearly three-fourths (74.0%) were under the age of 25–49 years of whom 58.0% were females and 0.5% of clients were new positive from age less than 5 years.

*“Priority for ICT testing was given for clients with high VL and new ART initiated by their partner and children. The positive yield on PLHIV partners and children was high. However, some of the ART clients were not voluntary to elicit their contacts for testing because some of them were feared their contacts were HIV positive and feared disclosing their positive result to their spousal or no-spousal partners. Others had no time to bring their contacts for HIV testing in working days. Hence, by the decision of the health facility and sub-city management, we gave services on Saturday, Sunday and holidays to increase and address index case testing services though the guidelines do not recommend serving on Saturday and Sunday”* [A 39 years old female ART focal].

**Table 5:** Positivity yield category by Service Delivery Point (SDP) and population category from October 1, 2017, to September 30, 2019, at the 90-90-90 targets of surge project in health centers of Lideta sub-city, Addis Ababa, Ethiopia

Service delivery points (SDPs)	Number of HIV tested	Number of identified positives	Yield (%)
TB	312	26	8.3
Adult OPD	12,638	122	0.96
Dermatology	516	2	0.4
Paediatrics OPD(< 5Years)	614	0	0
Cough OPD	704	18	2.6
Emergency OPDs	1026	24	2.3
Index Case Testing (ICT)	676	33	4.9
ANC	3662	26	0.7
VCT	5772	146	2.5
KP	438	7	1.6
SNS	45	2	4.4
Total	26,403	406	1.5
**Identified tested-positive people by population category**			
Female commercial sex worker	610	19	3
Long distance driver	219	11	5
Mobile worker	4574	78	1.7
Prisoner	30	2	6.7
Children of people living with HIV	479	12	2.5
Partners of people living with HIV	274	21	7.7
Orphan and vulnerable children (OVC)	15	1	6.7
Other MARPs	6776	97	1.4
General population	13,426	165	1.2
Total	26,403	406	1.5

Yield=(Number of tested / Number of Positives) *100

The overall performance of ART initiation (second 90) for positive clients in the last two years was 54.0% of whom half (50.0%) were in the same-day ART (treatment upon diagnosis).

Moreover, the overall ART initiation against the newly identified positives was 86.0%. Despite all newly identified positive people expected to be enrolled on ART treatment, only 78.0% of new positive people were enrolled on ART in the health centers, 8.0% were initiated into other health facilities by confirmed referrals, 1.2% died and 13.0% declined for ART initiation. Furthermore, 13.5% of new positive people were referred to other public health facilities, private facilities, and local NGOs for ART initiation for the last two years.

In this evaluation, it is shown that the overall retention rate of ART-initiated clients was 80.0% which demonstrated that 20.0% of ART-initiated clients in the health centers dropped their treatment for the last two years (Table 6). Furthermore, the overall score of the fidelity dimension was 84.7% (Table 7).

*“HIV-positive clients were refused for starting ART. This was the reason for low performance in this regard. The possible reason that HIV-positive clients could not start their medication was fear of disclosing their status, needing more time, not believing in their positive result, giving the wrong address, and choosing religious treatments like wholly water and praying for God to be cured of this virus. Although HIV-positive clients started ART, the majority of them lost their follow-up after one or more months. Hence, ensuring those ART clients who were not initiated for ART were true new positives or not is our big challenge “*[A 39 years old female ART focal from health centers].

**Table 6:** Judgment analysis matrix of compliance dimension for the evaluation of the 90-90-90 targets of surge project in health centers of Lideta sub-city, Addis Ababa, Ethiopia, 2020

Indicators	E	O	W	S	A (%)	JP
The proportion of SDPs performed HIV testing according to national guidelines for the last two years	46	46	10.3	10.3	100	V.good
Percentage of people who received HIV testing according to national guidelines for the last two years	26,403	26,367	10.2	10.2	100	V.good
The proportion of new HIV-positive adults and children enrolled in HIV care according to national guidelines for the last two years	316	316	11.5	11.5	100	V.good
The proportion of adults and children enrolled in HIV care received CTX prophylaxis based on their eligibility according to national guidelines for the last two years	88	88	8.3	8.3	100	V.good
The proportion of adults and children enrolled in HIV care and eligible for IPT who took at least one dose of isoniazid preventive therapy (IPT) according to national guidelines for the last two years	407	366	7.2	6.3	88.0	Good
The proportion of adults and children newly enrolled in HIV care and tested for viral load according to national guidelines for the last two years	297	245	12.5	10.3	82.0	Good
The proportion of HIV-positive clients referred to other health facilities for ART initiation according to national guidelines for the last two years	33	26	9.5	7.5	79.0	Good
Percentage of HIV-positive adults and children referred to the health centers for ART initiation according to the national guideline for the last two years.	55	55	7.5	7.5	100	V.good
Percentage of clients assessed for HIV testing using risk assessment tools based on guidelines	21	12	12.5	7.1	57.0	poor
Percentage of clients assessed and screened during ART visit time based on guidelines	21	18	10.5	9.0	86.0	Good
Overall compliance score			100	88.0	88.0	Good

E: Expected, O: Observed, W: weight, S: Score= ((observed X weight)/Expected), A: Achievement in percentage= ((S/W) * 100), JP: Judgement Parameter

**Table 7:** Judgment analysis matrix of fidelity dimension for the evaluation of the 90-90-90 targets of surge project in health centers of Lideta sub-city, Addis Ababa, Ethiopia, 2020

Indicators	E	O	W	S	A (%)	JP
Percentage of new positive adults and children who received ART care on the same day for the last two years	462	232	10	3.3	33.0	Poor
Percentage of adults and children who received viral load tests for the last 12 months	3060	3060	10	10	100	V.good
The proportion of new positive adults and children who received ART care and retained on treatment for the last two years	448	359	8	6.4	80.0	Good
Percentage of performance meetings conducted for the last two years	360	302	10	8.4	84.0	Good
Percentage of feedback documented for the last two years	156	136	8.5	7.4	87.1	Good
Percentage of reports documented in the health facility for the last two years	384	378	9	8.9	98.9	V.good
Percentage of completeness for each variable in the patient chart, ART register, and Smart Care-ART in the summary sheet	417	404	7.5	7.3	97.3	V.good
Percentage of validity for each variable in the patient chart, ART register, and Smart Care-ART in the summary sheet	417	409	6.6	6.5	98.5	V.good
Percentage of consistency for each variable in the ART register and SmartCare-ART in the summary Sheet	417	400	7.6	7.3	96.1	V.good
The proportion of providers with a positive attitude toward project implementation	216	210	8.0	7.8	97.5	V.good
Percentage of ART Provider-client interaction properly conducted during follow-up date	231	205	8.2	7.3	89.0	V.good
Percentage of provider-client interaction properly provided during risk assessment in OPDs	105	60	6.5	3.7	56.9	Poor
Overall fidelity score			100	84.7	84.7	Good

E: Expected, O: Observed, W: weight, S: Score= ((observed X weight)/Expected), A: Achievement in percentage= ((S/W) * 100), JP: Judgement Parameter

This evaluation showed that the overall viral load testing coverage performance was 100.0% with a suppression rate of 95.0% (third 90). Viral load was tested outside the implementing health centers in Addis Ababa’s central regional laboratory.

*“In every six months, we had done viral load tests for our ART patients. According to the laboratory records, greater than 90.0% of ART clients were received viral load-suppressed results. Since the viral load was tested in the Addis Ababa regional laboratory, the turning around time sometimes was high and some results were not returned. However, we were achieving the third 90 targets and we are trying to enhance the performance over”* [A 55 years old male ART focal].

**Fig. 2:**
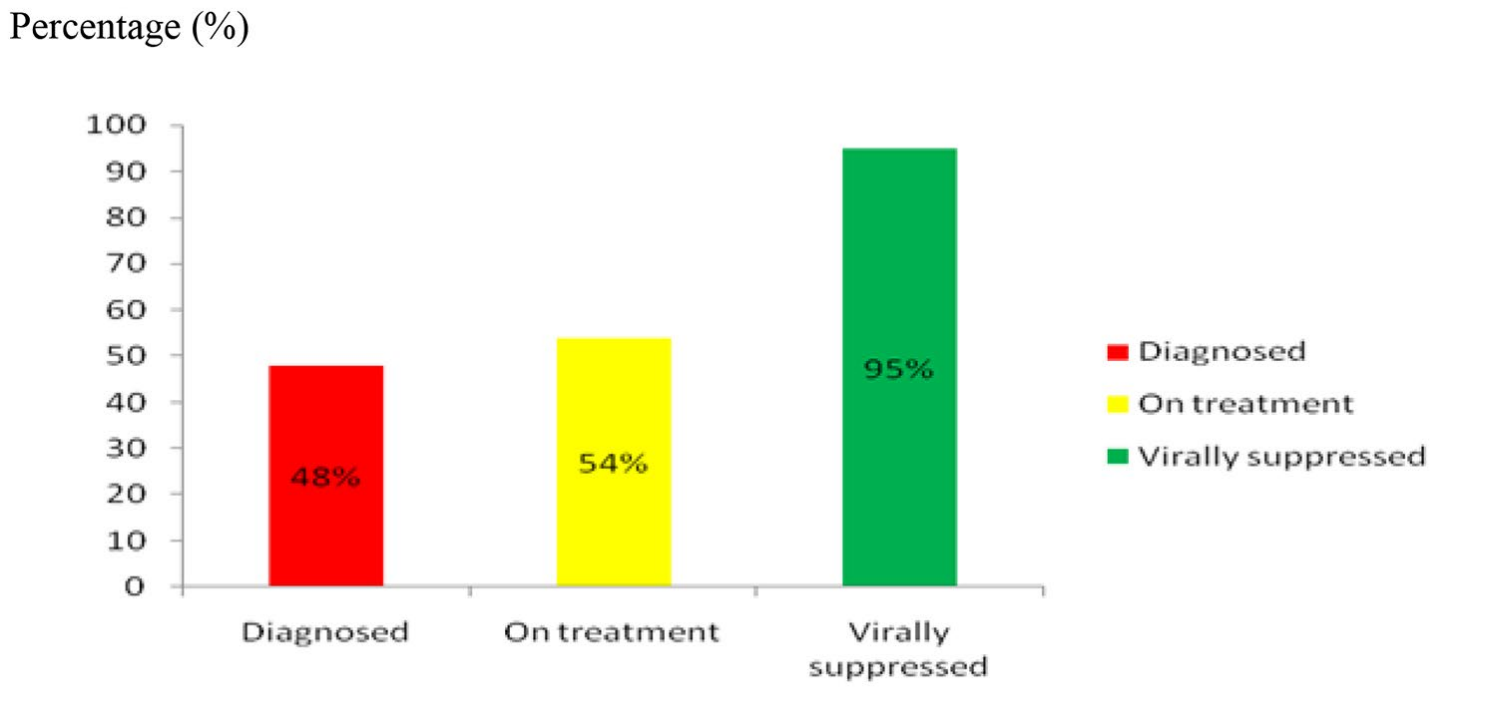
Diagnosis, treatment and viral load suppression status among clients in the surge project

Moreover, this evaluation showed that 48.0% of all people living with HIV had known their HIV status, 54.0% of all people diagnosed as HIV infected were received sustained antiretroviral therapy and 95.0% of all people receiving antiretroviral therapy had viral suppression against the given target in Lideta sub-city (Fig. 2).

Percentage (%).

### Client satisfaction with ART services

#### Description of ART clients

A total of 419 participants were *responded to the interviewer-administered questionnaire* with a response rate of 99.0%. More than three-fourths of the participants were between 25 and 49 years with a mean age of 38 years (SD = 11.078). The majority (44.9%) of participants were married and 99.5% of participants were residents of Addis Ababa. Furthermore, 64.2% were females and 34.4% were secondary school level (Table 8).

**Table 8:** Socio-demographic characteristics of participants of the 90-90-90 targets of surge project in health centers of Lideta sub-city, Addis Ababa, Ethiopia, 2020 (n = 419)

Variables	Frequency	Percent (%)
Age		
15–24 25–49	32 327	7.6 78
≥ 50	60	14.4
Sex of participants		
Male	150	35.8
Female	269	64.2
Marital status of participants		
Single	117	27.9
Married	188	44.9
Widowed	52	12.4
Divorced	62	14.8
Address of participants		
Addis Ababa	398	95.0
Out of Addis Ababa	21	5.0
Educational level of the participants		
Unable to read and write	37	8.8
Able to read and write	69	16.5
Primary school	115	27.4
Secondary school	144	34.4
College and above	54	12.9
Occupational status of participants		
Unemployed	79	18.9
Governmental employee	76	18.1
Self-employee	135	32.2
Mobile worker/Daily labour	46	11.0
Merchant	83	19.81
Monthly income (Ethiopian Birr)		
Less than 600	135	32.2
601–1650	104	24.8
1651–3200	90	21.5
3201–5250	58	13.8
5251–7800	24	5.7
≥ 7801	8	2.0

The finding of this evaluation revealed that the overall satisfaction of ART clients towards the accommodation of the 90-90-90 targets of the surge project in the Lideta sub-city was 89.3%. Moreover, the satisfaction of ART clients on card room service delivery and appropriateness of services hours was 56%, and 80% % respectively (Table 9).

**Table 9:** Judgment analysis matrix of accommodation dimension for the evaluation of 90-90-90 targets of surge project in health centers of Lideta sub-city, Addis Ababa, Ethiopia, 2020 (n = 419)

Indicators	E	O	W	S	A (%)	JP
The proportion of clients who perceived that appropriate hours of ART services are set	419	327	8.2	6.6	80.0	Good
The proportion of ART clients satisfied with the convenience of waiting rooms	419	360	9.8	8.4	86.0	Good
The proportion of ART clients who perceived that waiting time is appropriate	419	351	7.5	6.3	84.0	Good
The proportion of ART clients who perceived that a convenient appointment system is set	419	415	6.5	6.4	99.0	Good
The proportion of ART clients satisfied with the total time spent to get services was right	419	325	7.3	5.8	80.0	Good
The proportion of ART clients satisfied with the convenience of the providers’ understanding, focus, and close to them	419	416	9.7	9.6	99.0	V.good
The proportion of ART clients satisfied with the convenience of the providers’ effort of understanding to their health status	419	416	10.4	10.3	99.0	V.good
Proportion ART clients are satisfied with the convenience of the providers’ responses to their questions	419	413	7.6	7.5	98.0	V.good
The proportion of clients satisfied with the service delivery of pharmacy staff	419	407	8.5	8.2	97.0	V.good
Percentage of clients satisfied with service delivery of laboratory staff	419	390	7.3	6.8	93.0	V.good
The proportion of clients satisfied with the service delivery of card room staff	419	235	7.7	4.3	56.0	Poor
Overall score of accommodation			100	89.3	89.3	Good

E: Expected, O: Observed, W: weight, S: Score= ((observed X weight)/Expected), A: Achievement in percentage= ((S/W) * 100), JP: Judgement Parameter

This evaluation revealed that the overall implementation level of the process of the 90-90-90 targets of the surge project was 89.5% with an aggregate score of 95.8%, 88.0%, 84.7%, and 89.3% for availability, compliance, fidelity, and accommodation dimensions respectively and judged as “needs improvement” based on the predetermined judgment criteria.

#### Factors associated with ART client satisfaction

In the multivariable logistic regression analysis, ART clients whose ages between 15 and 24 were 78% less likely to be satisfied than ART clients whose ages were 50 years and above (AOR: 0.22, 95% CI: 0.08, 0.69), married ART clients were 53% less likely to be satisfied than divorced ART clients (AOR: 0.47, 95% CI: 0.25, 0.87). Moreover, the government-employed ART clients were 74% less likely to be satisfied compared to merchant ART clients (AOR: 0.26, 95% CI: 0.12, 0.54) with ART services (Table 10).

**Table 10:** Bivariable and multivariable logistic regression analysis for factors associated with the satisfaction of clients on ART services of 90-90-90 targets of surge project in health centers of Lideta sub-city, Addis Ababa, Ethiopia, 2020 (n = 419)

Variables	Satisfaction category		COR	AOR (95% CI)
	Dissatisfied n (%)	Satisfied n (%)		
Age				
15–24	20(4.8)	12(2.9)	0.32	0.22 (0.08, 0.69)*
25–49	177(42.2)	150(35.8)	0.46	0.60 (0.32, 1.13)
≥ 50	21(5.0)	39(9.3)	1	1
Sex				
Male	88(21.0)	62(14.8)	0.66	0.78 (0.51, 1.22 )
Female	130(31.0)	139(33.2)	1	1
Marital status				
Single	59(14.2)	58(13.8)	0.66	0.82(0.42, 1.62)
Married	114(27.2)	74(17.7)	0.44	0.47 (0.25, 0.87)*
Widowed	20(4.8)	32(7.6)	1.08	0.97 (0.44, 2.17)
Divorced	25(5.9)	37(8.8)	1	1
Occupation				
Unemployed	33(7.9)	46(10.9)	0.96	1.14 (0.54, 2.45)
Governmental Employee	56(13.4)	20(4.8)	0.24	0.26 (0.12,0.54)*
Self-employee	69(16.5)	66(15.8)	0.66	0.72 (0.38, 1.36)
Mobile workers	36(8.6)	34(8.1)	0.65	0.65 (0.31, 1.35)
Merchant	24(5.7)	35(8.3)	1	1

## Discussion

The overall implementation status of the 90-90-90 targets of the surge project was 89.5% which was judged as “needs improvement” based on the predetermined judgment criteria. The finding of this study was higher than the study conducted in Ethiopia which reported that the overall quality of the services was 73.3% [24]. This discrepancy might be due to differences in the availability of resources and the provision of services per the recommended protocol. Moreover, the implementation level of availability, compliance, fidelity, and accommodation dimensions was 95.8%, 88%, 84.7%, and 89.3% respectively.

This evaluation has briefed that 48.0% of all people living with HIV were known their HIV status, 54.0% of those diagnosed with HIV infection were received antiretroviral therapy and 95.0% of all people receiving antiretroviral therapy were had viral suppression in the Lideta sub-city. In this perspective, this evaluation finding contrasts with the Ethiopian national guideline of comprehensive HIV prevention, care, and treatment [25] which recommends that 90% of all people living with HIV should know their HIV status, 90% of those diagnosed with HIV infection should receive sustained antiretroviral therapy and 90% of all people receiving antiretroviral therapy irrespective of immune status should have viral suppression.

In this evaluation, healthcare providers’ training status on comprehensive ART service was as per the desired standard. This finding is in line with the Ethiopian Federal Ministry of Health (FMoH) national guidelines for comprehensive HIV prevention, care, and treatment, the human resource distribution guide [26]. The availability of sufficiently trained healthcare providers in these vital healthcare service areas will enable healthcare workers to deliver quality and holistic treatment services for HIV patients.

Likewise, comprehensive HIV counselling and testing (VCT), viral load sample collection and testing, and PITC were fully covered by trained health care providers which was consistence with the requirement of the Ethiopian national consolidated guidelines for comprehensive HIV prevention, care, and treatment [27]. Essential drugs like co-trimoxazole suspension were stocked out in the last six months in all health centers. On the contrary, pertinent program drugs such as OI drugs and ARV drugs were in stock for the last six months. The availability of those drugs would have a positive impact on the viral suppression effort done in the sub-city.

Furthermore, guidelines were available in ART, PMTCT, and VCT clinics. The availability of these guidelines enables healthcare providers to adhere to the recommended care and treatment services. The findings of this evaluation showed that job aids like Printers, Photocopy machines, and Laptops were found to be not availed in the last six months in the health centers of the surge project. This implies that the shortage of these aids has a significant negative impact on supporting their activities with an integrated documentation system.

Moreover, the overall availability score of the 90-90-90 targets of the surge project was 95.8% which was judged as “very good”. This evaluation finding showed that all of the ART clients were screened and assessed based on the follow-up form. Furthermore, measuring clients’ weight, assessing WHO staging, assessing opportunistic infections, and assessing ARV adherence status were observed to be practised as per the recommended protocol. This finding was supported by the Ethiopian national guidelines for comprehensive HIV prevention, care, and treatment protocol guidelines [25].

Likewise, healthcare providers adhered fully to screening co-trimoxazole preventive therapy, assessing patients’ ARV adherence, and setting HIV prevention plans. This finding was consistence with the UNAIDS protocol which recommends that all HIV patients have to be screened for co-trimoxazole preventive therapy and assessed for ARV adherence [28].

In this evaluation, only 19.0% and 85.7% of ART clients were assessed for adverse drug effects (ADEs) and pregnancy status respectively. This has a serious implication that healthcare providers should assess adverse drug effects (ADEs) to prevent further complications.

In the direct observation of risk assessment utilization, healthcare providers have assessed two-thirds of clients whether ever been tested for HIV, occupational risks, and clinical signs and symptoms of HIV/AIDS. Furthermore, assessing TB and sexual risk factors of clients were the least practice which was 38.0%. This implies that there is a higher chance of further transmission of HIV unless they do not have an effort in this regard.

In this evaluation, the proportion of clients who took Isoniazid preventive therapy (IPT), tested for viral load suppression, and assessed for HIV testing using risk assessment tools were 88%, 82%, and 57% respectively. Moreover, this evaluation showed that 88.0% of the 90-90-90 targets of the surge project were implemented according to the national standards which was judged as “good”.

In this evaluation, the proportion of people diagnosed with HIV infection who received antiretroviral therapy was 54.0%. This finding was higher than the UNAIDS Global AIDS report which showed that the coverage of antiretroviral therapy was 46% [29] and was lower than the other study conducted in Botswana where 73% of people diagnosed with HIV infection had received antiretroviral therapy [30].

This evaluation findings showed that 48.0% of all people living with HIV were known their HIV status in the Lideta sub-city. This study was lower than the study conducted in New York City which revealed that Latinos (Hispanics) and blacks have achieved that all people living with HIV were known their HIV status [31]. This discrepancy might be due to differences in the use of risk assessment tools and the commitment of healthcare providers. This finding was also lower than the other population-based survey which was conducted in Botswana where 83% of people living with HIV were known their HIV status [32]. This implies that providing awareness creation to the community regarding the benefit of knowing the HIV status was not provided as adequately as possible in the evaluation area.

In this evaluation, it was also shown that 95.0% of all people receiving antiretroviral therapy had viral suppression against the given target. This finding was relatively higher than the study conducted in New York City which showed that whites had achieved viral suppression by 80% [31]. This variation might be due to the difference in the strengthened follow-up of clients not to drop out of their treatment.

This evaluation showed that the overall performance of the 90-90-90 targets regarding the fidelity dimension was 84.7% which was judged as “good”. Moreover, the proportion of clients who were tested for their HIV status and tested for viral load suppression was 100%. Likewise, the proportion of performance meetings held, percentage of feedback documented, and percentage of completeness of patient documents were 84%, 87%, and, 97% respectively. Furthermore, the percentage of validity of variables in the patient chart, and the proportion of providers with a positive attitude on the project implementation was 98% and 97% respectively.

This evaluation has also revealed that the proportion of clients who received cotrimoxazole prophylaxis, and new positive clients who received ART were 88% and 86% respectively.

The accommodation of the 90-90-90 targets of surge project ART services provided by health centers of the Lideta sub-city was found to be 89%. This finding was almost similar to a study conducted in the Jima zone, Ethiopia which showed that the accommodation of ART service was 89.64% [33]. This consistency might be due to the availability of a similar healthcare delivery system. Moreover, the satisfaction of ART clients on the adequacy of working hours, convenience of the waiting room, and waiting time was 91.6%, 85.4%, and 83.5% respectively. This finding was higher than the study conducted in Gondar, Ethiopia which showed that the satisfaction of ART clients on the adequacy of working hours, convenience of the waiting room, and waiting time was 84%, 71%, and 85% respectively. This difference might be due to differences in the organization’s structural set-up to be convenient for the clients.

Likewise, 95.9%, 97.6%, and 56% of ART clients were satisfied with the convenience of the counseling room, the convenience of the providers’ response to their questions, and the service delivery by card room workers. Furthermore, more of the clients were satisfied with the delivery of laboratory and pharmacy services. This implies that there is a well-integrated service delivery in each pharmacy and laboratory service units.

Moreover, ART clients aged 15–24 were 78% less likely to be satisfied with the accommodation of ART services of the 90-90-90 targets of the surge project than ART clients whose ages were 50 years and above (AOR: 0.22, 95% CI: 0.08–0.69). This may be due to adolescents in this age group might need additional youth-friendly services provision and this might not be sufficiently availed to ensure their needs. Another important predictor of client satisfaction was marital status. Married ART clients were 53% less likely to be satisfied than divorced ART clients (AOR: 0.47, 95% CI: 0.25–0.87). Furthermore, government-employed ART clients were 74% less likely to be satisfied compared to merchant ART clients (AOR: 0.26, 95% CI: 0.12 − 0.54) with ART services. This study was consistence with the study conducted in the Shoa zone, Oromia, Ethiopia where merchants were 2.6 times more likely to be satisfied with the overall services compared to government employees [34].

### Strengths and limitations of the evaluation

The strength of the evaluation was that triangulation was used to get accurate, and consistent results. Moreover, the evaluation used four dimensions to evaluate the process of the 90-90-90 targets of the surge project to address a wide area of performance aspects and employed both qualitative and quantitative methods to get more accurate and detailed results.

The limitations of this evaluation were that Hawthorn’s effect during the direct observation might have contributed to the relatively high-performance scores of the provider during provider-client interaction. To minimize this, the first three observations were dropped to bring the provider to his/her actual behaviour.

Social desirability bias which might result in relatively high levels of satisfaction with the service was the other possible limitation.

Proper training of the data collectors on proper interview techniques and interviewing clients in a separate room were efforts taken to minimize it.

Information bias during key informant interviews was also another possible limitation. To minimize it, rapport building was done.

## Conclusion

The availability of resources for the 90-90-90 targets of the surge project was found to be “needs improvement”. Moreover, the compliance, fidelity, and accommodation dimensions needed improvement. Shortage of manpower, test kits, INH drugs, and viral load testing machines were found to be deficient. Furthermore, provider-client interaction and clients’ satisfaction with the service provision of card rooms were found to be poor.

Moreover, this evaluation revealed that clients’ awareness of their HIV status was low. Likewise, there were low rates of treatment coverage which showed that there was poor achievement in the 1st and the 2nd 90s.

Furthermore, age 15–24, being married, and being government-employed were factors negatively associated with ART client satisfaction. Therefore, the health centers should improve the quality of risk assessment utilization, and capacity building, strengthen target-focused testing, and maximize case detection by minimizing the missed opportunity of eligible clients for HIV testing.

Moreover, the Addis Ababa Regional Health Bureau should strengthen supportive supervision and ensure the availability of sufficient resources continuously like test kits, drugs, and reagents. Furthermore, the evaluators had better conduct outcome and impact evaluations by taking this evaluation finding as a baseline.

## Data Availability

All the data were included in the study, and data will be available upon reasonable request from the corresponding author.

## Abbreviations

AIDS: Acquired Immunodeficiency Syndrome
ART: Antiretroviral Therapy
CDC: Centers for Diseases Control
EDHS: Ethiopia Demographic and Health Survey
HC: Health Center
HIV: Human Immunodeficiency Virus
HMIS: Health Management Information System
OPD: Out Patient Department
OVC: Orphans and Vulnerable Children
PLHIV: People Living with HIV
PMTCT: Prevention of Mother-to-Child Transmission
SOPs: Standard Operating Procedures
SPSS: Statistical Package for Social Science
STI: Sexual Transmitted Infection
TB: Tuberculosis
UNAIDS: United Nations Programme on HIV and AIDS
VL: Viral Load
WHO: World Health Organization
